# Self-Esteem and Oral Health-Related Quality of Life within a Cleft Lip and/or Palate Population: A Prospective Cohort Study

**DOI:** 10.3390/ijerph18116078

**Published:** 2021-06-04

**Authors:** Antonia Aleksieva, Giacomo Begnoni, Anna Verdonck, Annouschka Laenen, Guy Willems, Maria Cadenas de Llano-Pérula

**Affiliations:** 1 Department of Oral Health Sciences-Orthodontics, KU Leuven and Dentistry, University Hospitals Leuven, Kapucijnenvoer 7, Blok A, Bus 7001, 3000 Leuven, Belgium; antonia.aleksieva92@gmail.com (A.A.); giacomo.begnoni@uzleuven.be (G.B.); an.verdonck@uzleuven.be (A.V.); guy.willems@kuleuven.be (G.W.); 2 Interuniversity Institute for Biostatistics and Statistical Bioinformatics, KU Leuven and University Hasselt, 3000 Leuven, Belgium; annouschka.laenen@kuleuven.be

**Keywords:** orthodontics, self-esteem (SE), cleft lip and/or palate, oral health, OHRQoL, CPQ, IOTN, OASIS, motivation, patient’s expectations

## Abstract

(1) Objective: To investigate the oral health-related quality of life (OHRQoL) and self-esteem (SE) of a population with cleft lip and/or palate (CLP) and to compare it with a non-affected control cohort. (2) Materials and methods: This study comprised 91 CLP patients and a control group of 790 individuals, seeking orthodontic treatment. OHRQoL and SE were assessed by the Child’s Perception Questionnaire (CPQ) and the Dutch adaptation of the Harter’s Self-Perception Profile for Adolescents. Treatment need and self-perception of oral aesthetic were assessed using the Index of Orthodontic Treatment Need (IOTN) and the Oral Aesthetic Subjective Impact Scale (OASIS). Patients’ expectations and motivation for treatment were also scored. Linear models were used for statistical comparisons between groups. (3) Results: The cleft group scored higher in all domains of the CPQ, OASIS, IOTN and regarding SE for the domains of scholastic competence, athletic competence, physical appearance and behavioral conduct. The cleft group was not only more motivated and expected less discomfort during treatment but also had higher expectations for the treatment outcome. (4) Conclusions: The OHRQoL of CLP patients is strongly correlated with the presence of an oral cleft, while SE remains a personal resource not influenced by the malocclusion or medical condition.

## 1. Introduction

Clefts are the most common congenital anomaly in the orofacial region. Their etiology is still unclear: it is believed that both genetic and environmental factors are involved. The prevalence of cleft lip with or without cleft palate (CLP) varies between the different ethnicities and is higher in the Asian population, followed by Caucasian and African populations [1]. CLP occurs more frequently in boys than in girls and the prevalence of this condition varies around 1.7 per 1000 live births [2] with left-sided clefts being twice as common as right-sided clefts [3].

Quality of life (QOL) is a contemporary topic that describes the general well-being of a population, including negative and positive life features. The health-related QOL (HRQoL) evaluates the QOL in its relationship with health. The oral health-related quality of life (OHRQoL) has been defined as “The impact of oral disorders on aspects of everyday life that a patient or person values, that are of sufficient magnitude, in terms of frequency, severity or duration to affect their experience and perception of life” [4].

Self-esteem (SE) can be defined as the perception of one′s ability to effectively deal with the surrounding environment, and it is affected by the reactions of others towards an individual [5]. There is no clear evidence that an orthodontic treatment improves self-esteem [6] which has been reported to be a stable construct by several researchers in the field [7]. The observation of SE during adolescence can stimulate further research regarding the possible factors that can affect it. Moreover, whether oral clefts do or do not negatively affect the SE is not yet clearly stated.

The impact of malocclusion and orthodontic treatment on the OHRQoL of non-affected individuals has been well-documented [8,9,10]. Since treatment of orofacial clefts is multidisciplinary and extends from birth to early adulthood, impact of this condition on the patient’s OHRQoL is expected. However, comparative studies with large cleft populations and adequate control groups are still lacking, especially assessing patients’ expectations and motivation for treatment.

The aim of this study is to investigate OHRQoL, SE, orthodontic treatment need, expectations and motivation for orthodontic treatment in a CLP population and to compare it with a healthy control group before regular orthodontic treatment with fixed appliances.

## 2. Materials and Methods

### 2.1. Study Population and Selection Criteria

This research is an observational prospective cohort study that evaluates the OHRQoL and the SE of CLP patients and compares it with a non-affected control group. The study protocol was approved by the medical ethical committee of University Hospitals Leuven and KU Leuven University with registration number S51642 (ML5739). Written informed consent was obtained from all participants prior to data collection.

For the control group, all patients with no prior history of orthodontic treatment presenting for a first consultation at the Department of Orthodontics of University Hospitals Leuven between 2009 and 2018 were invited to participate. The cleft group was recruited during the consultations at the same Department or at the multidisciplinary Cleft Team between 2017 and 2020. All types of cleft lip and/or palate were included.

For both groups, adolescents with syndromes, mental retardation or psychiatric problems, patients who were undergoing or had undergone orthodontic treatment with fixed appliances, adolescents who did not understand Dutch or those whose parents or caregivers did not understand Dutch were excluded. Before completing the questionnaire, the patients in the control group were asked whether they had any medical base pathology and, if so, they were also excluded. It is important to mention that all bilateral and unilateral cleft lip and palate patients had undergone early orthodontic treatment (transversal expansion of the upper jaw with removable appliance prior to the secondary alveolar bone graft (SABG)). Some of the cleft palate (CP) and cleft lip (CL) patients had also undergone early maxillary expansion to solve transversal problems. None of the patients in the control group had ever had an orthodontic appliance of any kind.

All patients were asked to complete the questionnaires at one time point, prior to the start of orthodontic treatment with fixed appliances. Participants (and parents/caregivers since participants were minor) were approached and asked if they wanted to participate in the study. The purpose of the research was explained and informed consent was given to be read and signed. They also received a short explanation on how to fill in the questionnaire. Participants could take all the time they needed to answer the questions on the spot or could also take the questionnaires home and return them when ready.

### 2.2. Objective and Perceived Orthodontic Treatment Need

Orthodontic treatment need was determined by the Index of Orthodontic Treatment Need (IOTN). Different calibrated orthodontic residents scored the objective orthodontic treatment need by using the Dental Health Component (DHC) and the patient evaluated his/her own orthodontic treatment need by using the Aesthetic Component (AC). DHC of 3 or greater and AC of at least 5 were considered as indicating the need for orthodontic treatment [11].

### 2.3. Oral Health Related Quality of Life

The OHRQoL of the patients was scored with the Dutch translation of the Child Perception Questionnaire (CPQ_11–14_). This questionnaire consists of 37 questions divided into 4 subdomains: functional limitations, oral symptoms, social well-being and emotional well-being. Beside each domain, rated separately, a total CPQ score is also obtained. All questions refer to the frequency of occurrence of a given phenomenon in the last 3 months, related to lips, teeth and jaws. A lower CPQ score translates as a better OHRQoL.

Self-perception of oral esthetics was assessed with the Oral Aesthetic Subjective Impact Scale (OASIS), consisting of five questions concerning the perceptions of others and themselves of oral aesthetics, answered on a 7-point Likert scale. A higher score indicates a higher concern of the malocclusion. The results of the OASIS were extended by the perceived need for orthodontic treatment, the Aesthetic Component (AC) of IOTN.

### 2.4. Self-Esteem

The Dutch adaptation of the Harter’s Self-Perception Profile for Adolescents (SPPA), called “Competentie Belevingsschaal voor Adolescenten” (CBSA), was used to assess the patient’s self-esteem (SE). CBSA consists of 35 questions evaluating the adolescent’s self-perception in different domains: scholastic competence, social acceptance, athletic competence, physical appearance, behavioral conduct, close friendship and global self-worth. Items are scored on a 4-point scale and recoded, higher numbers representing positive self-perception. By using the age norms of the Dutch adaptation of the SPPA, we converted the percentile scores of the raw results [12]. The questionnaire is composed in a specific way, largely to decrease the possibility that answers are guided by social desirability. The CBSA questionnaire has been previously tested for validity and retested for stability [12,13].

### 2.5. Expectations of Treatment

A sample of questions regarding patient’s expectations of orthodontic treatment was selected from the tool of Sayers and Newton [14] and translated to evaluate the expectations during and after treatment, as well as the expected reactions of family and friends (Supplementary Materials), answered on a 7-point Likert scale. A higher score for the first four questions was associated with more discomfort during the treatment. Six questions were used to score post-treatment expectations and one question scored expected entourage acceptance.

### 2.6. Motivation for Treatment

“Motivation for treatment stems from several sources and can be defined as the conscious or unconscious stimulus for action toward a desired goal” [15]. To assess patient’s motivation for orthodontic treatment, the following two questions were asked: ‘Are you motivated to wear an orthodontic appliance?’ and ‘Do you think that your family and friends are going to support you?’, answered on a 7-point Likert scale. A higher score meant more motivation for treatment and more support from family and friends.

### 2.7. Statistical Analysis

Linear models were used for statistical comparisons between groups, correcting for age and gender, which is especially relevant due to the gender differences between groups. Post-hoc comparisons with Tukey correction for multiple testing between the different cleft types were used. Results are presented as mean differences between groups with a 95% confidence interval. All tests are two-sided, and a 5% significance level was adopted. Analysis was performed using SAS software (version 9.4 for Windows).

## 3. Results

Sample description is presented in Table 1. Based on the sample size, the study obtained 80% of power for showing group differences assuming small to medium effect sizes (Cohen’s D = 0.32).

In the cleft group, there was a significant difference in gender distribution (32.97% females and 67.03% males, while 51.01% females and 48.99% males in the control group). Both the gender and cleft type distribution were expected since they are inherent to this condition [16].

Table 2 shows the results of IOTN. With this index, DHC is always scored as 5 in CLP patients. Since the control group is selected from an orthodontic population, the results of IOTN give an idea of the severity of the preexisting malocclusion.

Table 3 shows the results of OHRQoL in both groups. Cleft patients scored high for al domains of CPQ, which means a lower OHRQoL than non-affected patients. The differences between both groups are significant for the domains of functional limitations, emotional functioning, social functioning and the total CPQ score. The only domain that does not significantly differ between the two groups is oral symptoms. The OASIS scale, which was used to estimate the patient’s self-perception of dental aesthetics, scored without the AC, was not significantly different between the groups. However, when adding the AC, results were significantly higher for the cleft group, meaning a worse level of dental attractiveness according to themselves.

Self-esteem did not differ significantly between both groups, except for the domains of behavioral conduct and scholastic competence, which show a higher self-esteem in the cleft group. Only three of the SE domains (social acceptance, close friendship and global self-worth) were higher within the control group, but differences were not significant.

The expectations of discomfort during treatment were significantly higher for the control group, while expectations of the outcome were lower compared to those of the cleft group. The motivation for orthodontic treatment was much higher in the cleft group compared with controls.

The comparison of OHRQoL, self-esteem, motivation and expectations of the different cleft types did not yield any significant difference, except for the oral symptoms, functional restrictions and the AC component of IOTN, where bilateral cleft patients had worse scores compared to cleft palate and cleft lip (Table 4, Table 5, Table 6 and Table 7). As expected, the bilateral cleft group had the lowest OHRQoL. No significant differences were noted amongst cleft types regarding SE.

## 4. Discussion

The present study assesses the possible differences in OHRQoL and SE between cleft patients and non-affected individuals. Although OHRQoL of CLP patients has already been investigated [17,18,19,20,21], this is, to the best of our knowledge, the first research assessing SE, expectations and motivation for orthodontic treatment in patients with this condition compared with non-affected individuals.

Previous research investigating OHRQoL in pediatric cleft patients is somewhat contradictory. Some studies [22] found no significant differences in children with and without CLP, while others found a significant difference only for the domain of oral symptoms, despite the higher DHC in the cleft group [20], which we did not find. These contradictions may be due to differences in sample size, age and gender distribution and/or study population (orthodontic or not), as well as the use of different questionnaires. For instance, the study samples of Wogelius et al. [22] and Ward et al. [20] were both smaller than our CLP (75 and 36 respectively compared with 91 in our study) and control group (75 vs. our 790). While Ward et al. used the COHIP questionnaire to assess OHRQoL, Wogelius et al. used CPQ. CPQ (11–14) has found to be a reliable tool to evaluate OHRQoL in cleft and non-cleft patients [23,24,25], reason why we choose to use it. Although we could have included patients before early expansion treatment (6 to 9 years old), we consciously chose to focus on patients after this treatment since CPQ is only validated for subjects between 11–14 years old, and in younger patients, questionnaires are often answered by the parents, which may introduce a bias.

The self-perceived aesthetic and orthodontic treatment need, scored by the OASIS and AC photographs, was higher for the cleft group, but differences were only significant for the AC. It is important to remark that the cleft group has not been compared with the general population, but with a control group of patients seeking orthodontic treatment, with an average orthodontic treatment need indicated by a score of 4 for the AC and 3 for the DHC (moderate need for orthodontic treatment [26]). In the literature, higher levels of treatment need have been correlated with a lower OHRQoL, although this correlation has only been found to be significant for the domains of emotional and social wellbeing [27,28]. However, comparing cleft patients with an ideal group without treatment need would always yield differences.

Regarding SE, cleft patients scored higher than controls in the domains of scholastic competence, athletic competence, physical appearance and behavioral conduct. The control group scored higher in social acceptance, close friendship and global self-worth which may be interpreted as SE not depending on the malocclusion or oral disorders but more on the personal characteristics, as supported by other studies [29,30]. The presence of more male subjects in the cleft group could possibly explain the higher SE results in this population for the domain of Scholastic competence, as it has been reported that girls have tendency to underestimate themselves for it [31]. To avoid these effects to a certain extent, results were statistically corrected for gender.

Regarding treatment expectations, patients in the control group expected to find more discomfort during treatment than cleft patients, which can be explained by the large medical experience of the cleft group. This can also explain the higher score for the expected positive reaction and support from the family/friends found in the cleft group, as they are already familiar with previous medical treatments and have received information as to what to expect from long term treatment of their condition by different specialists. This could also be linked with cleft patients having higher expectations of treatment results than non-affected patients. Due to the often severe skeletal and dental malocclusions of cleft patients, it is not always possible to reach the same standard of treatment outcome as within non-affected individuals. Missing teeth in the front area, the long treatment time and the decrease of motivation by the patients can also lead to compromised results. It is therefore important that clinicians provide accurate information to cleft patients and parents, to avoid unrealistic expectations that could ultimately affect the patient’s QoL.

In addition, we assessed motivation for treatment with two questions. It is important to note that this assessment was not based on a validated tool. This was an intended decision, aimed to avoid results being influenced by patients having to answer many different and extensive questionnaires [32]. We found the cleft group to be more motivated than the control group, which could be related to more negative self-perceived aesthetics [33].

The comparison of the different cleft types showed more negative results in the BCLP group for all domains of OHRQoL, OASIS and AC. This initially contradicts the results of Crepaldi et al. [34], who found the type of cleft not to affect the general health-related quality of life. This could be explained by the smaller sample size (57 patients) and the fact that comparisons were made between ‘single’ (CP and CL) and ‘complex’ (UCLP and BCLP) clefts. This could have masked the individual differences of each group.

This study has certain limitations to take into consideration. The previous information of cleft patients regarding orthodontic treatment could have influenced the results. Patients were also exclusively selected from the University Hospitals Leuven, Belgium and no patients from private practice were included. In spite of some aspects of our sample distribution being different between groups, the cleft sample in our study is in accordance with the prevalence reported in Belgium for this condition: males are generally more affected than females [16]. However, we believe that the mean age of the two groups (12,74 and 12,76 for control and cleft group) is similar enough to allow comparisons, as it is already known that SE (and OHRQoL) are age- and gender-related factors [30,35]. The sample size of both the cleft and control groups is quite large, even with the relatively low prevalence of oral clefts. We were able to assess SE and could confirm its independence from the presence of an oral cleft. The comparison of the motivation and expectations between the two groups gives an idea of the needed measurements to improve cooperation of the patients and to facilitate the orthodontic treatment.

## 5. Conclusions

The main findings of the present study suggest that:

- OHRQoL depends on the degree of malocclusion.
- SE, contrariwise, is a stable, personal-depending factor, which should be taken into consideration when engaging in treatment of CLP patients.
- CLP patients showed a lower OHRQoL than controls in all domains, except for oral symptoms.
- The control group showed lower SE scores for scholastic competence (Sc), athletic competence (Ac), physical appearance (Pa) and behavioral conduct (Bc).
- Cleft patients were more motivated for treatment and expected better results than non-affected individuals.
- The experience of the cleft group within their treatment course may have influenced their perception of orthodontic treatment and the possibilities around it.
- Patients with bilateral CLP scored worse than other cleft types for the domain of oral symptoms, functional restrictions and the AC component of IOTN. No other significant differences were observed within the cleft types.

## Figures and Tables

**Table 1:** Descriptive information of the sample.

Variable	Statistic	Control	Cleft	*p*-Value
	N	790	91	
**Age**	Mean	12.76	12.74	0.488
	Std	1.26	1.86	
	Median	13	12	
	IQR	(12.00; 14.00)	(11.00; 14.00)	
	Range	(10.00; 16.00)	(10.00; 17.00)	
**Gender**				0.001 *
Female	*n*/*N* (%)	403/790 (51.01%)	30/91 (32.97%)	
Male	*n*/*N* (%)	387/790 (48.99%)	61/91 (67.03%)	
**Type of cleft**				
UCLP	*n*/*N* (%)		36/91 (39.56%)	
BCLP	*n*/*N* (%)		13/91 (14.29%)	
Cleft palate	*n*/*N* (%)		26/91 (28.57%)	
Cleft lip	*n*/*N* (%)		16/91 (17.58%)	

Variables presented with % are analyzed using chi-square tests. The remaining variables are analyzed using a Mann–Whitney U test, * represents significant *p*-value. Abbreviations: UCLP: unilateral CLP; BCLP: bilateral CLP.

**Table 2:** IOTN.

Variable	Statistic	Control Group	Cleft Group
AC	N	779	90
	Mean	4.01	4.69
	Std	1.98	3.09
	Median	4.00	4.00
	IQR	(3.00; 5.00)	(2.00; 8.00)
	Range	(1.00; 10.00)	(1.00; 10.00)
DHC	N	781	
	Mean	3.28	
	Std	1.042	
	Median	3	
	IQR	(2.00; 4.00)	
	Range	(0.00; 5.00)	

Abbreviations: AC: aesthetic component, DHC: dental health component.

**Table 3:** Cleft versus control group.

Concept	Variable	MD (95% CI)	*p*-Value
**OHRQoL (CPQ)**	Oral symptoms	0.459 (−0.18; 1.10)	0.160
	Functional limitations	2.277 (1.45; 3.11)	<0.001 *
	Emotional well-being	2.323 (1.17; 3.48)	<0.001 *
	Social well-being	3.968 (3.02; 4.92)	<0.001 *
	Total CPQ score	8.351 (5.53; 11.17)	<0.001 *
**OASIS/AC**	OASIS without AC	0.884 (−0.19; 1.96)	0.108
	AC	1.142 (0.64; 1.64)	<0.001 *
**Self-esteem (CBSA)**	RS_Sc	0.947 (0.13; 1.77)	0.024 *
	RS_Sa	−0.397 (−1.01; 0.21)	0.202
	RS_Ac	0.054 (−0.74; 0.85)	0.894
	RS_Pa	0.176 (−0.53; 0.88)	0.624
	RS_Bc	0.889 (0.18; 1.60)	0.014 *
	RS_Cf	−0.135 (−0.75; 0.47)	0.663
	RS_Gs	−0.064 (−0.70; 0.57)	0.844
**Expectation**	Expectations during treatment	−1.276 (−2.50; −0.05)	0.041 *
	Expectation acceptance of the surrounding	0.343 (0.02; 0.67)	0.040 *
	Expectation of the treatment outcome	4.779 (2.91; 6.65)	<0.001 *
**Motivation**	Motivation patient	0.707 (0.31; 1.11)	0.001 *
	Support from family and friends	0.672 (0.32; 1.03)	<0.001 *

CI: confidence interval, MD: mean difference >0: higher score for cleft group, * represents significant *p*-value. Abbreviations: OHRQoL: Oral Health-Related Quality of Life; CPQ: Child Perceptions Questionnaire; OASIS: Oral Aesthetic Subjective Impact Score; AC: aesthetic component; CBSA—Competentie-Belevingsschaal voor Adolescenten; RS_Sc: scholastic competence; RS_Sa: social acceptance; RS_Ac: athletic competence; RS_Pa: physical appearance; RS_Bc: behavioral conduct; RS_Cf: close friendship; RS_Gs: global self-worth.

**Table 4:** OHRQoL within the different types of cleft.

Groups	1. Oral Symptoms		2. Functional Limitations		3. Emotional Well-Being		4. Social Well-Being		5. Total CPQ Score	
Comparison	MD	*p*-Value	MD	*p*-Value	MD	*p*-Value	MD	*p*-Value	MD	*p*-Value
	(95% CI)		(95% CI)		(95% CI)		(95% CI)		(95% CI)	
Global test		0.037		0.037		0.291		0.245		0.076
BCLP	1.590	0.417	3.559	0.171	1.794	0.881	3.155	0.576	10.098	0.398
vs UCLP	(−1.11; 4.29)		(−0.94; 8.06)		(−4.57; 8.16)		(−3.29; 9.60)		(−6.69; 26.89)	
BCLP	1.569	0.520	5.224	0.037 *	3.421	0.584	5.277	0.221	15.490	0.137
vs CL	(−1.43; 4.56)		(0.23; 10.22)		(−3.63; 10.47)		(−1.863; 12.42)		(−3.12; 34.10)	
BCLP	3.113	0.026 *	4.496	0.068	4.314	0.333	4.161	0.377	16.084	0.086
vs CP	(0.28; 5.95)		(−0.23; 9.22)		(−2.36; 10.99)		(−2.60; 10.92)		(−1.53; 33.70)	
UCLP	−0.022	1.000	1.665	0.742	1.627	0.897	2.122	0.804	5.392	0.815
vs CL	(−2.61; 2.56)		(−2.64; 5.97)		(−4.46; 7.75)		(−4.04; 8.29)		(−10.67; 21.46)	
UCLP	1.523	0.222	0.937	0.891	2.520	0.528	1.006	0.950	5.986	0.614
vs CP	(−0.54; 3.59)		(−2.50; 4.38)		(−2.34; 7.38)		(−3.91; 5.93)		(−6.84; 18.81)	
CL	1.544	0.457	−0.728	0.975	0.893	0.983	−1.116	0.970	0.593	1.000
vs CP	(−1.20; 4.29)		(−5.30; 3.85)		(−5.57; 7.36)		(−7.66; 5.43)		(−16.46; 17.65)	

CI: confidence interval; MD: mean difference > 0: higher score for first category; post-hoc comparisons with Tukey correction for multiple testing, * represents significant *p*-value. Abbreviations: BCLP—cilateral CLP; UCLP—unilateral CLP; CL—cleft lip; CP—cleft palate.

**Table 5:** OASIS/AC.

Groups	OASIS without AC		AC	
Comparison	MD (95% CI)	*p*-Value	MD (95% CI)	*p*-Value
Global test		0.063		0.005
BCLP vs. UCLP	2.802 (−2.16; 7.77)	0.454	2.371 (−0.24; 4.98)	0.088
BCLP vs. CL	5.622 (0.12; 11.12)	0.043	2.662 (−0.23; 5.55)	0.082
BCLP vs. CP	3.672 (−1.54; 8.88)	0.258	3.802 (1.07; 6.54)	0.003 *
UCLP vs. CL	2.820 (−1.930; 7.57)	0.409	0.291 (−2.20; 2.79)	0.990
UCLP vs. CP	0.870 (−2.92; 4.66)	0.931	1.431 (−0.56; 3.42)	0.243
CL vs. CP	−1.950 (−6.99; 3.09)	0.742	1.140 (−1.51; 3.79)	0.673

CI: confidence interval; MD: mean difference > 0: higher score for first category; post-hoc comparisons with Tukey correction for multiple testing, * represents significant *p*-value. Abbreviations: OASIS—Oral Aesthetic Subjective Impact Score; AC—aesthetic component; BCLP—bilateral cleft lip and palate; UCLP—unilateral cleft lip and palate; CL—cleft lip; CP—cleft palate.

**Table 6:** Self-Esteem (CBSA).

Groups	1. RS_Sc		2. RS_Sa		3. RS_Ac		4. RS_Pa		5. RS_Bc		6. RS_Cf		7. RS_Gs	
Comparison	MD	*p*-Value	MD	*p*-Value	MD	*p*-Value	MD	*p*-Value	MD	*p*-Value	MD	*p*-Value	MD	*p*-Value
	(95% CI)		(95% CI)		(95% CI)		(95% CI)		(95% CI)		(95% CI)		(95% CI)	
Global test		0.839		0.649		0.948		0.866		0.470		0.125		0.951
BCLP vs. UCLP	0.491	0.977	−0.251	0.994	0.572	0.967	0.264	0.995	0.692	0.899	0.277	0.993	0.016	1.000
	(2.68; 3.66)		(2.86; 2.36)		(2.66; 3.82)		(2.61; 3.14)		(1.92; 3.30)		(2.44; 2.99)		(2.95; 2.98)	
BCLP vs. CL	0.301	0.996	−1.092	0.756	0.113	1.000	−0.582	0.964	1.703	0.416	−2.034	0.295	−0.515	0.977
	(3.22; 3.82)		(3.99; 1.80)		(3.49; 3.71)		(3.77; 2.61)		(1.19; 4.59)		(5.05; 0.98)		(3.80; 2.77)	
BCLP vs. CP	1.072 (2.26; 4.40)	0.833	−0.863 (3.60; 1.88)	0.842	0.122 (3.29; 3.53)	1.000	−0.160 (3.18; 2.86)	0.999	0.973 (1.76; 3.71)	0.788	−0.453 (3.30; 2.40)	0.976	0.134 (2.98; 3.24)	1.000
						
UCLP vs. CL	−0.191	0.998	−0.841	0.814	−0.459	0.980	−0.846	0.852	1.012	0.713	−2.311	0.100	−0.530	0.961
	(3.23; 2.85)		(3.34; 1.66)		(3.57; 2.65)		(3.60; 1.91)		(1.48; 3.51)		(4.91; 0.29)		(3.37; 2.31)	
UCLP vs. CP	0.581	0.923	−0.612	0.853	−0.451	0.964	−0.424	0.957	0.281	0.983	−0.729	0.794	0.118	0.999
	(1.84; 3.00)		(2.61; 1.38)		(2.93; 2.03)		(2.62; 1.77)		(1.71; 2.27)		(2.80; 1.35)		(2.15; 2.38)	
CL vs. CP	0.772	0.923	0.229	0.996	0.008	1.000	0.421	0.982	−0.731	0.888	1.582	0.441	0.648	0.942
	(2.45; 4.00)		(2.42; 2.88)		(3.29; 3.31)		(2.50; 3.34)		(3.38; 1.92)		(1.18; 4.34)		(2.36; 3.66)	

CI: confidence interval; MD: mean difference > 0: higher score for first category; post-hoc comparisons with Tukey correction. Abbreviations: CBSA—Competentie-Belevingsschaal voor Adolescenten; RS_Sc—scholastic competence; RS_Sa—social acceptance; RS_Ac—athletic competence; RS_Pa—physical appearance; RS_Bc—behavioral conduct; RS_Cf—close friendship; RS_Gs—global self-worth; BCLP—Bilateral CLP; UCLP—Unilateral CLP; CL—cleft lip, CP—cleft palate.

**Table 7:** Expectations and Motivation.

Groups	Expectations during Treatment		Expected Entourage Acceptance		Expected Treatment Outcome		Patient Motivation		Motivation Others	
Comparison	MD	*p*-Value	MD	*p*-Value	MD	*p*-Value	MD	*p*-Value	MD	*p*-Value
	(95% CI)		(95% CI)		(95% CI)		(95% CI)		(95% CI)	
Global test		0.074		0.897		0.323		0.469		0.639
BCLP	2.077	0.853	0.291	0.947	0.390	0.999	−0.526	0.823	0.219	0.975
vs UCLP	(−4.71; 8.86)		(−1.10; 1.68)		(−7.12; 7.90)		(−2.12; 1.07)		(−1.15; 1.59)	
BCLP	1.741	0.930	0.373	0.921	5.058	0.389	−1.037	0.419	−0.310	0.95
vs CL	(−5.78; 9.26)		(−1.17; 1.92)		(−3.27; 13.38)		(−2.80; 0.73)		(−1.83; 1.21)	
BCLP	−3.008	0.686	0.111	0.997	1.189	0.979	−0.347	0.948	−0.198	0.984
vs CP	(−10.12; 4.11)		(−1.35; 1.57)		(−6.69; 9.07)		(−2.02; 1.32)		(−1.63; 1.24)	
UCLP	−0.335	0.999	0.082	0.999	4.668	0.329	−0.511	0.816	−0.529	0.715
vs CL	(−6.83; 6.16)		(−1.25; 1.41)		(−2.52; 11.86)		(−2.04; 1.01)		(−1.84; 0.78)	
UCLP	−5.085	0.056	−0.180	0.971	0.799	0.983	0.179	0.980	−0.417	0.723
vs CP	(−10.26; 0.09)		(−1.24; 0.88)		(−4.94; 6.54)		(−1.04; 1.40)		(−1.46; 0.63)	
CL	−4.750	0.278	−0.262	0.962	−3.869	0.547	0.690	0.679	0.112	0.997
vs CP	(−11.64; 2.14)		(−1.68; 1.15)		(−11.50; 3.76)		(−0.93; 2.31)		(−1.28; 1.50)	

CI: confidence interval, MD: mean difference > 0: higher score for first category; post-hoc comparisons with Tukey correction. Abbreviations: BCLP—bilateral CLP; UCLP—unilateral CLP; CL—cleft lip; CP—cleft palate.

## Data Availability

The dataset supporting the conclusions of this article is included within the article and its additional files. The detailed data of the children analyzed during the study is available from the corresponding author on reasonable request.

## Supplementary Materials

The following are available online at https://www.mdpi.com/article/10.3390/ijerph18116078/s1.
