# Supplementary material for: Self-Esteem and Oral Health-Related Quality of Life within a Cleft Lip and/or Palate Population: A Prospective Cohort Study
Source: Int J Environ Res Public Health. 2021 Jun 4;18(11):6078. doi: 10.3390/ijerph18116078 (PMC8200197; doi:10.3390/ijerph18116078)
Supplement: Supplementary file 1 [file ijerph-18-06078-s001.zip › ijerph-1201568-supplementary.pdf]

Annex 1. Translated questions from the Sayer-Newton expectations questionnaire

47. Denk je dat het dragen van een beugel **pijnlijk** zal zijn?

Zeker niet	1	2	3	4	5	6	7	Zeker wel

48. Denk je dat je door een beugel **problemen** zal ondervinden bij **spreken**?

Zeker niet	1	2	3	4	5	6	7	Zeker wel

49. Denk je dat je door een beugel **problemen** zal ondervinden bij **eten**?

Zeker niet	1	2	3	4	5	6	7	Zeker wel

50. Denk je dat je door een beugel **minder** dingen zal kunnen **eten of drinken**?

Zeker niet	1	2	3	4	5	6	7	Zeker wel

51. Hoe denk je dat andere mensen op je beugel zullen **reageren**?

Negatieve reactie	1	2	3	4	5	6	7	Positieve reactie

52. Verwacht je dat je **door** de orthodontische **behandeling**:

a) **rechtere tanden** zal hebben?

Zeker niet	1	2	3	4	5	6	7	Zeker wel

b) een **mooiere glimlach** zal hebben?

Zeker niet	1	2	3	4	5	6	7	Zeker wel

c) beter zal kunnen **eten**?

Zeker niet	1	2	3	4	5	6	7	Zeker wel

d) beter zal kunnen **spreken**?

Zeker niet	1	2	3	4	5	6	7	Zeker wel

e) gemakkelijker je tanden zal kunnen **poetsen**?

Zeker niet	1	2	3	4	5	6	7	Zeker wel

f) meer **zelfvertrouwen** zal krijgen?

Zeker niet	1	2	3	4	5	6	7	Zeker wel
